# Occupational Exposures to Organic Solvents and Asthma Symptoms in the CONSTANCES Cohort

**DOI:** 10.3390/ijerph18179258

**Published:** 2021-09-02

**Authors:** Guillaume Sit, Noémie Letellier, Yuriko Iwatsubo, Marcel Goldberg, Bénédicte Leynaert, Rachel Nadif, Céline Ribet, Nicolas Roche, Yves Roquelaure, Raphaëlle Varraso, Marie Zins, Alexis Descatha, Nicole Le Moual, Orianne Dumas

**Affiliations:** 1 Université Paris-Saclay, UVSQ, Univ. Paris-Sud, Inserm, Équipe d’Épidémiologie respiratoire intégrative, CESP, 94807 Villejuif, France; guillaume.sit@inserm.fr (G.S.); benedicte.leynaert@inserm.fr (B.L.); rachel.nadif@inserm.fr (R.N.); nicolas.roche@aphp.fr (N.R.); raphaelle.varraso@inserm.fr (R.V.); alexis.descatha@inserm.fr (A.D.); orianne.dumas@inserm.fr (O.D.); 2 Herbert Wertheim School of Public Health and Human Longevity Science & Scripps, Institution of Oceanography, UC San Diego, La Jolla, CA 92093, USA; noemie.letellier@inserm.fr; 3 Santé publique France Direction Santé Environnement Travail, 94415 Saint-Maurice, France; yuriko.IWATSUBO@santepubliquefrance.fr; 4 Population-Based Epidemiological Cohorts Unit, INSERM UMS 11, 94807 Villejuif, France; marcel.goldberg@inserm.fr (M.G.); celine.ribet@inserm.fr (C.R.); marie.zins@inserm.fr (M.Z.); 5 Faculty of Medicine, University of Paris, 75006 Paris, France; 6 APHP Centre—Université de Paris, Hôpital et Institut Cochin, Service de Pneumologie, 75014 Paris, France; 7 UNIV Angers, CHU Angers, Univ Rennes, Inserm, EHESP, Irset (Institut de recherche en santé, environnement et travail)—UMR_S1085, F-49000 Angers, France; yves.roquelaure@univ-angers.fr

**Keywords:** current asthma, asthma symptom score, occupational exposure, solvents, job-exposure matrix, population-based cohort

## Abstract

Solvents are used in many workplaces and may be airway irritants but few studies have examined their association with asthma. We studied this question in CONSTANCES (cohort of ‘CONSulTANts des Centres d’Examens de Santé’), a large French cohort. Current asthma and asthma symptom scores were defined by participant-reported respiratory symptoms, asthma medication or attacks, and the sum of 5 symptoms, in the past 12 months, respectively. Lifetime exposures to 5 organic solvents, paints and inks were assessed by questionnaire and a population-based Job-Exposure Matrix (JEM). Cross-sectional associations between exposures and outcomes were evaluated by gender using logistic and negative binomial regressions adjusted for age, smoking habits and body mass index. Analyses included 115,757 adults (54% women, mean age 47 years, 9% current asthma). Self-reported exposure to ≥1 solvent was significantly associated with current asthma in men and women, whereas using the JEM, a significant association was observed only in women. Significant associations between exposures to ≥1 solvent and asthma symptom score were observed for both self-report (mean score ratio, 95%CI, women: 1.36, 1.31–1.42; men: 1.34, 1.30–1.40) and JEM (women: 1.10, 1.07–1.15; men: 1.14, 1.09–1.18). Exposure to specific solvents was significantly associated with higher asthma symptom score. Occupational exposure to solvents should be systematically sought when caring for asthma.

## 1. Introduction

Asthma is the most reported work-related respiratory disorder. About 15% of adult asthma cases are caused by occupational exposures [1], and 20% of adults with asthma have experienced aggravation of their symptoms at work [2,3]. More than 400 occupational agents have been identified as asthmagens. They can be categorized as sensitizers (e.g., flours, latex, diisocyanates) and irritants [4,5,6]. According to the causative substance and the pathogenic mechanism, occupational asthma is classified as sensitizer-induced asthma or irritant-induced asthma [4,7,8]. The first type involves an immunologic mechanism and appears after a latency period [8]. The second type corresponds to asthma caused by non-immunological irritant mechanisms, which have not been precisely identified yet. Irritant-induced asthma appears after a single acute exposure to very high levels of irritants (reactive airway dysfunction syndrome (RADS), or Brooks’ syndrome) or may occur with a delayed onset after repeated low-dose exposures to irritants [8,9,10,11].

Solvents are volatile chemicals that can reach the respiratory system and have both an irritating and a sensitizing potential. As such, they might cause or aggravate asthma [12,13]. Solvents are widely used to produce paints and varnishes, as well as in printing activities, dry cleaning and cleaning products, or to degrease metals [14]. In France, in 2013, about 15% of workers were exposed to solvents [15]. Recently, their use has been more rigorously regulated due to increasing knowledge about their carcinogenicity and toxicity [16,17]. Regarding lung health, some studies reported associations between exposure to solvents and respiratory symptoms [18], chronic obstructive pulmonary disease (COPD) [19] or a decrease in lung function [20,21,22]. Until now, there is limited evidence on the specific association between occupational exposures to solvents and asthma [23,24,25,26,27]. In the late 1990s, case reports in the United Kingdom identified solvents as probable etiological agents of RADS [5,28]. Studies by Torén et al. (Swedish asthma case-control study), LeVan et al. (Singaporean cohort), Cakmak et al. and Saygun et al. (gun factory workers in Turkey) suggested an association between exposures to solvents and asthma but these studies focused only on general exposures to the vapor of solvents [23,24,25,26].

Altogether, few studies have considered the impact of occupational exposures to solvents on asthma and to the best of our knowledge none of them investigated specific solvents. Exposures to solvents at work are multifaceted and heterogeneous. To better identify risks factors for asthma, specific solvents or family of solvents should be considered. Therefore, we examined the association between occupational exposures to specific solvents and asthma in a large French population-based cohort by using two exposure assessment methods based on self-report and a population-based Job Exposure Matrix (JEM).

## 2. Materials and Methods

The CONSTANCES study is a French general population-based cohort [29]. Between 2012 and 2020, 202,045 participants aged 18–69 years were recruited in 24 health screening centers across France. The source population of CONSTANCES included salaried workers, professionally active or retired and their family (>85% of the French population) [29]. At enrolment, self-administered questionnaires were sent to participants to collect data including lifestyle, health, and occupations. Participants also had to attend a health examination in a Health Prevention Center (‘Centres d’examens de santé’) [29]. Our analyses are based on baseline data available in early 2020. At this time, 131,057 participants had ever worked in relevant jobs, of whom 115,757 had all the data needed (asthma, smoking status, Body Mass Index (BMI)) for our analyses (Figure S1).

Occupational exposures to organic solvents were assessed by self-report and a population-based Job-Exposure Matrix (JEM). At inclusion, participants had to complete a questionnaire on occupational exposure to various agents, including solvents, and a questionnaire on their whole job history (start and end dates, business sector, partial or full-time job). Each occupation was coded according to the French national job coding (‘Profession et Catégories Socio-professionnelles’, PCS 2003; https://www.insee.fr/fr/information/2400059, accessed on 1 March 2021) and industries (‘Nomenclature d’Activités Française’, NAF; https://www.insee.fr/fr/information/2406147, accessed on 1 March 2021).

Lifetime exposures to five specific organic solvents: gasoline (for hand washing or dry cleaning of metals and textile), trichloroethylene, white spirit, cellulosic thinner, and formaldehyde, and to paints/varnishes, inks/dyes, and “other solvents” were evaluated. Participants who reported exposures were asked to provide information about start and end year of exposure to each solvent. Based on this information, we created three self-reported exposure variables. First, we classified participants as ever exposed to each solvent or not. Second, we classified participants as reporting current exposure (e.g., current workers exposed within the past 2 years), past exposure, or no exposure. For the latter variable, exposures with missing dates were excluded. Third, we generated a variable considering the number of exposures to solvents in 4 categories: no exposure, exposure to 1 solvent, 2 solvents or ≥3 solvents. Self-reported lifetime exposures to welding fumes and metals dusts were also evaluated.

We created a population-based JEM, for each gender separately, by crossing data from current self-reported occupational exposures to solvents and current job. The “exposure axis” of the JEM included 8 chemicals selected from the CONSTANCES questionnaires (excluding other solvents) and the “job axis” included 640 job codes (486 4-digits and 154 3-digits codes). The methods to develop the JEM were similar to those used in previous studies and are detailed in the online supplement [30,31]. Briefly, for each job, we determined the percentage of participants with self-reported current exposure to each solvent. Depending on the percentages of exposed participants, the JEM ranks exposures into 4 categories: no exposures (<5%), low probability of exposure (from 5% to 9.9%), moderate probability of exposure (from 10% to 19.9%) and high probability of exposure (≥20%). Then, we applied the JEM to the current job to evaluate current exposures, and to full job histories to evaluate lifetime exposures. For the latter, if a participant had several jobs considered as exposed to a given solvent, the highest exposure level was retained. We also used the JEM to inform a binary classification distinguishing between “ever exposed” (combining low, moderate and high probability of exposure) and “never exposed” participants. Analyses of specific solvents, excluding other solvents, were conducted using this binary JEM.

Asthma was evaluated by a standardized questionnaire (Figure S2). We used standardized definitions of asthma similar to those previously used in ECRHS (European Community Respiratory Health Survey) or EGEA (Etude épidémiologique des facteurs Génétiques et Environnementaux de l’Asthme, l’hyperactivité bronchique et l’atopie) [32,33]. Ever asthma was defined by a positive answer to the question “have you ever had asthma?” and current asthma was defined among participants with ever asthma by the report in the past 12 months of symptoms, or use of an asthma treatment or at least an asthma attack. As previously described and validated [34,35], the asthma symptom score, ranging from 0 to 5, was defined as the sum of 5 asthma symptoms reported in the past 12 months [36]: breathless while wheezing, woken up with chest tightness, attack of shortness of breath at rest, attack of shortness of breath after exercise, and woken by attack of shortness of breath.

Associations between lifetime occupational exposures to solvents and current asthma were estimated by logistic regressions. For associations between solvents and asthma symptom score, we used negative binomial regressions to account for over-dispersion [34,37]. All analyses were adjusted for age, smoking status (non-smoker, former smoker or current smoker) and BMI in 3 categories (<25, 25–29.9, ≥30 kg/m^2^). All analyses were stratified by gender and interaction tests were performed. In the main analyses, we used lifetime occupational exposures from the questionnaire and the JEM. The reference category for exposures corresponded to participants never exposed to any of the solvents during their occupational life. For a given specific exposure, we compared exposed participants to the reference category, excluding from the analysis participants exposed to other solvents. The sample size for each analysis differed according to the exposure assessment method (questionnaire or JEM) and availability of the outcome (current asthma or asthma symptom score, Figure S1). In additional analyses, we examined current occupational exposure evaluated by both methods. In analyses of current asthma, we also evaluated associations according to age at asthma onset (<16 or ≥16 years). In analyses of asthma symptom score, we also analyzed associations stratified by asthma status. Finally, we performed a sensitivity analysis excluding all participants exposed to welding fumes and metal dusts (potential co-exposures). We used SAS 9.3 (SAS Institute Inc., Cary, NC, USA) for all analyses.

## 3. Results

### 3.1. Description of the Study Population

At the time of data extraction, complete data were available for 115,757 adults participating in CONSTANCES (Supplement Figure S1). Participants with missing data for smoking and BMI were similar to included participants; participants with missing data for asthma were slightly older, more often smokers, overweight and exposed to solvents (Table S1). Women represented 54% of the sample and the mean age was 47 years; 13.6% of the participants reported ever asthma and 9.6% current asthma. For the asthma symptom score, 12.7% of women and 11.4% of men had a score ≥ 2 (Table 1).

Men reported exposure to at least one solvent more frequently than women (25.1% vs. 11.7%). Paints/varnishes and formaldehyde were the exposures most frequently reported by men and women, respectively. Among participants ever exposed to solvents, 31% were also exposed to welding fumes or metal dusts.

### 3.2. Occupational Exposures to Solvents and Current Asthma

**Figure 1:**
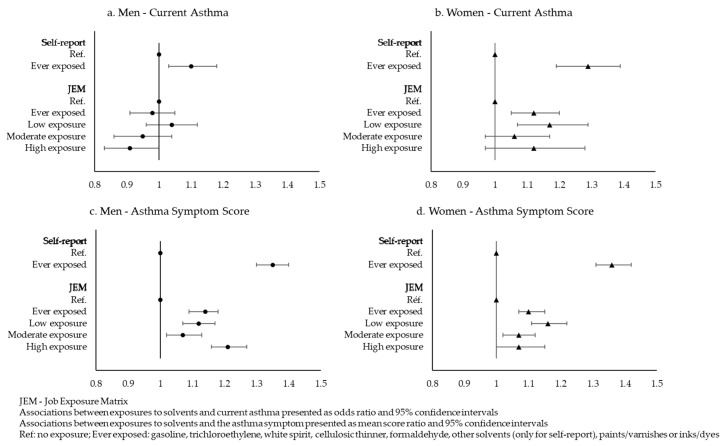
Association between lifetime exposures to at least one solvent assessed by self-report or JEM and current asthma or asthma symptom score.

Using self-report, we found a positive and significant association between lifetime exposure to at least one solvent and current asthma (adjusted OR 1.10, 95% CI 1.03–1.18 in men; 1.29, 1.19–1.39 in women, Figure 1a,b).

Regarding specific exposures, most studied solvents were positively associated with current asthma in both genders (Table 2), except formaldehyde, which was not associated with asthma in men, and cellulosic thinner in both genders. Associations were stronger when the number of solvents was higher in both women and men. Associations were also higher in women than in men with a significant interaction for at least one solvent, gasoline, trichloroethylene, white spirit and paints. When excluding participants exposed to welding fumes and metal dusts, associations between lifetime exposure to solvents and current asthma were similar (Table S2).

When studying current exposures to solvents and current asthma (Table S3), we observed significant associations only with self-reporting in women. No significant association was observed in men regardless of the assessment method used.

When accounting for age at asthma onset among participants with current asthma (Table S4), with self-report, we observed a significant association between lifetime exposure to solvents and current adult-onset asthma in both men and women. For childhood-onset current asthma, a significant association was only observed in women, with a lower OR. When using the JEM, we observed no significant association between lifetime exposure to solvents and adult or childhood-onset asthma in men and significant associations for adult and children-onset asthma in women.

### 3.3. Occupational Exposures to Solvents and Asthma Symptom Score

With either method of assessment, lifetime exposure to solvents was associated with higher asthma symptom score in both men and women (Figure 1c,d). In analyses using the JEM with four exposure levels, we observed significant associations for all probabilities of exposure, but without a trend for higher exposure probabilities.

For specific solvents, we observed significant associations with both self-report and the JEM, although the strength of associations was less pronounced with the JEM. Self-reported lifetime occupational exposure to each solvent was significantly associated with a higher asthma symptom score in both men and women, with adjusted Mean Score Ratio (MSR) ranging from 1.23 to 1.71 in women and 1.33 to 1.58 in men (Table 2). For most solvents except formaldehyde, associations were slightly higher in women than in men but interactions were mostly not significant. With the JEM, we also observed significantly higher asthma symptom scores associated with exposure to all solvents except for formaldehyde in women (MSR 0.94, 95%CI 0.89–0.99) (Table 3). Significant interactions between gender and exposure were observed only for formaldehyde and ink/dyes. The mean score ratio ranged from 0.94 to 1.24 in women and from 1.10 to 1.26 in men. Considering the number of exposures to solvents, associations were stronger with higher number of exposures when using both self-report and the JEM (*p* for trend <0.001). Excluding participants exposed to welding fumes and metal dusts did not change the results (Table S2).

When examining current exposures to at least one solvent and asthma symptom score (Table S3), significant and borderline significant (*p* = 0.09) associations were observed with both self-report and JEM in men and women.

### 3.4. Stratified Analysis by Asthmatic Status

Analyses of asthma symptom score stratified by ever/never asthma status showed significant associations between exposures to at least one solvent and a higher asthma score regardless of the asthma status, with the two methods of exposure assessment (Table 4). The interaction between asthmatic status and solvents was significant in most of these analyses.

## 4. Discussion

In this very large French population-based cohort, we investigated exposures to specific solvents in relation to current asthma and asthma symptoms. We observed significant associations between lifetime self-reported exposures to solvents and current asthma in both women and men. Using the JEM, significant associations were observed only in women. We also observed modest but significant associations between lifetime occupational exposures to almost all solvents and a higher asthma symptom score in both women and men, whatever the method of exposure assessment used. These findings further strengthen the evidence supporting an impact of occupational exposures to solvents on asthma expression, providing important targets for primary and secondary prevention of asthma.

These results are consistent with those from Torén et al. and LeVan et al., which suggested that solvents were associated with adult-onset asthma [23,24]. Our findings are also consistent with a few studies on trichloroethylene and paints [16,25,38], which suggested an association between these chemicals and asthma or respiratory diseases. Associations between inks, which contained acrylates, and adult-onset asthma have also been reported [39]. Associations between formaldehyde, a known asthmagen, and asthma outcomes were not entirely consistent in our analyses, in particular in women. However, as formaldehyde is now classified as a human carcinogen by the International Agency for Research on Cancer (IARC) [40], its use has probably declined since 2012. To the best of our knowledge, occupational exposures to gasoline, cellulosic thinner and white spirit have never been studied in relation to asthma or respiratory diseases. This study is the first to show significant associations between several specific solvents and asthma and also the first to examine associations between occupational exposures to solvents and the asthma symptom score. Altogether, our results provide important additional evidence supporting the link between exposure to solvents, and the occurrence or aggravation of asthma in a professional environment [5,6,11,23,24]. However, we cannot identify underlying mechanisms nor speculate on irritant- versus sensitizer-induced processes. While solvents are known as potential respiratory irritants, occupational exposure to solvents has also been associated with allergic disorders such as allergic rhinitis or atopic dermatitis [6].

In our study, we used both self-report and a JEM to evaluate occupational exposures. Our results based on self-report are consistent with previous studies, which only used this assessment method [23,24]. In a previous analysis in CONSTANCES, significant associations were also observed between self-reported exposure to solvents and cognitive performance [17]. Self-report is one of the most common assessment methods but can introduce misclassification biases [31]. For example, participants may not precisely recall their past exposures. Participants with asthma may remember their exposures more precisely (differential misclassification bias), which would lead to an overestimation of associations. To address this issue, we built a population-based JEM with which the risk of differential misclassification is reduced [31]. This method, though, has non-differential misclassification, partly due to heterogeneity (tasks, work conditions) between subjects in the same job, and could underestimate associations between exposures and outcomes [41]. The JEM we developed also has its own limitations. We generated the JEM with data on current exposures and current jobs and then used this exposure assessment to evaluate lifetime exposures on complete job histories. Exposures in a given job can change over the years and current exposures may be different from former exposures in the same job [42]. Unexpectedly, we did not find higher associations in groups with the highest probability of exposure when using the JEM with 4 classes, which may suggest misclassification in assessment of level of exposure. We thus used the JEM with 2 exposures groups, which favored sensitivity. Interestingly, in the case of a highly prevalent exposure such as solvents, sensitivity has been suggested to be more important than specificity [41].

In our analyses, we used a standardized definition of asthma: current asthma and asthma symptom score. The continuous score, compared to a dichotomous definition of asthma, increases power to identify risk factors for asthma [34,35,43]. When studying occupational exposures, this score is particularly relevant because occupational asthma is underrecognized in clinical practice. Using the score may help with revealing and studying asthma including subjects not identified as asthmatics [4,34]. When we stratified analyses by lifetime asthma status, we found significant associations between exposure to solvents and asthma symptom score in participants both with or without asthma, suggesting that exposure to solvents may worsen respiratory health in all workers. Associations with exposures and outcomes differed for asthma symptom score and current asthma. Although these different estimates are not directly comparable (mean score ratios vs. odds ratios), we observed more significant associations between lifetime exposures to solvents and the asthma symptom score than with current asthma, notably when exposures were assessed with the JEM. This disparity could be explained by an under-recognition of work-related asthma [39]. Occupational exposure to solvents may also be associated with a specific asthma phenotype (e.g., irritant-induced asthma), which may not commonly be recognized as asthma [44]. Finally, a healthy worker effect may also contribute to the different results for asthma symptom score and current asthma. Current asthmatics may choose jobs without hazardous occupational exposures or modify their work conditions, to a greater extent than participants with symptoms but not identified as asthmatics, leading to an underestimation of associations between occupational exposure and current asthma [45].

Associations between exposure to solvents and current asthma also differed according to gender. When exposures were assessed by self-report, associations were higher in women than in men. With the JEM, associations with current asthma were significant only among women. When examining asthma symptom score, differences between men and women were less consistent. In previous studies, analyses were rarely stratified by gender. Torén et al. reported that associations between exposure to solvents and asthma were not different according to gender [23]. Alif and al. observed that exposure to solvents was associated with a greater lung function decline in women than in men [21]. LeVan et al. did not take into account gender and other studies only selected men [24,38]. Perception of exposures and symptoms may differ according to gender, which may lead to a gender-specific bias in exposure assessment in the current study [46]. In a study on factors modifying the strength of association between estimates of exposure provided by self-report and a JEM, the association was slightly higher in men suggesting that men had a better assessment of exposure [47]. Beyond biases, differences can be explained by actual differences in exposed jobs and tasks performed. Even within the same occupation, differences may exist in occupational patterns between women and men, and the assignment of some tasks is notably gendered [48]. However, this would indicate a greater level of exposure to solvents in women than in men. Different pathophysiological processes between women and men, and greater vulnerability of women to the same level of exposure, can also explain gender differences [49].

One strength of our study is the large size of the cohort, leading to a large power to detect even modest associations. CONSTANCES is also one of the only cohorts with the number of women exposed to specific solvents high enough to investigate association by gender. However, our study also has some weaknesses, including its cross-sectional design. In addition, we could not consider potential co-exposures to all known asthmagens, which were not available in CONSTANCES. However, when excluding participants exposed to welding fumes and metal dusts, associations between lifetime exposure to solvents and current asthma or asthma symptom score remained similar and significant, suggesting that our observed associations were not due to these specific asthmagens.

## 5. Conclusions

Occupational exposures to solvents were associated with a higher asthma symptom score in both men and women, whatever the assessment methods used. Associations were significant for most of the solvents studied, including gasoline, white spirit, trichloroethylene, cellulosic thinner, paints and inks. Regarding current asthma, associations were significant when estimating exposure with self-report in both men and women, and only among women when using the JEM. Our results suggest that exposure to solvents triggers respiratory symptoms. Since solvents are a common occupational exposure, their impact on respiratory health and particularly asthma may well be substantial. Clinicians should consider occupational exposures to solvents as a potential trigger or risk factor and recommend decreasing such exposures in patients with asthma-like symptoms. Further analyses investigating the underlying mechanisms would be helpful.

## Figures and Tables

**Table 1:** Description of the study population.

	Men N = 53,357	Women N = 62,400
Age, mean (Standard deviation)	47.6 (12.9)	46.8 (12.9)
BMI, kg/m^2^		
<25	49.0	64.9
25–29.9	38.7	23.5
≥30	12.3	11.6
Smoking habits		
Nonsmoker	41.3	50.9
Current smoker	20.1	17.8
Former smoker	38.7	31.3
Exposures to solvents (self-report)		
At least one solvent *	25.1	11.7
Gasoline	8.6	1.3
Trichloroethylene	8.2	1.4
White spirit	8.7	1.7
Cellulosic thinner	4.5	0.6
Formaldehyde	2.1	2.5
Other solvents	11.1	6.8
Paints/Varnishes	9.1	2.4
Inks/Dyes	2.5	1.5
Exposures to solvents (JEM)		
At least one solvent ⁑	57.8	22.3
Gasoline	38.2	4.6
Trichloroethylene	21.5	2.7
White spirit	37.4	6.0
Cellulosic thinner	24.5	1.8
Formaldehyde	8.9	8.1
Paints/Varnishes	45.9	8.3
Inks/Dyes	11.4	7.6
Ever asthma	13.6	13.6
Current asthma	9.2	9.9
Asthma symptom score		
0	70.5	66.9
1	18.1	20.4
≥2	11.4	12.7
Mean (Sd)	0.5 (0.9)	0.5 (1.0)

Results presented as percentages unless otherwise specified. BMI—body mass index; JEM—Job Exposure Matrix. * Exposure to at least one solvent (self-report): gasoline, trichloroethylene, white spirit, cellulosic thinner, formaldehyde, other solvents, paints/varnishes or inks/dyes, ⁑ Exposure to at least one solvent (JEM): gasoline, trichloroethylene, white spirit, cellulosic thinner, formaldehyde, paints/varnishes or inks/dyes.

**Table 2:** Association between self-reported lifetime occupational exposures to solvents and current asthma or asthma symptom score.

	Current Asthma					Asthma Symptom Score				
		Men (*n* = 46,786)		Women (*n* = 54,157)		Men (*n* = 49,206)		Women (*n* = 56,483)		
	*n*	Odds Ratio (95% CI)	*n*	Odds Ratio (95% CI)	*p* Inter	*n*	Mean Score Ratio (95% CI)	*n*	Mean Score Ratio (95% CI)	*p* Inter
Non-exposed (ref.)	34,040	1	47,196	1		35,816	1	49,216	1	
At least one solvent *	12,746	1.10 (1.03–1.18)	6961	1.29 (1.19–1.39)	0.001	13,390	1.34 (1.30–1.40)	7267	1.36 (1.31–1.42)	0.75
1 solvent	5547	1.03 (0.93–1.13)	4567	1.27 (1.15–1.39)	0.003	5850	1.22 (1.16–1.29)	4770	1.29 (1.23–1.36)	0.28
2 solvents	3039	1.07 (0.95–1.22)	1530	1.25 (1.07–1.47)		3190	1.32 (1.23–1.41)	1596	1.41 (1.30–1.53)	
3 or more solvents	4160	1.24 (1.11–1.38)	864	1.46 (1.20–1.78)		4350	1.52 (1.44–1.61)	901	1.62 (1.46–1.80)	
*p-trend*		0.0002		<0.0001			<0.0001		<0.0001	
Gasoline	4367	1.14 (1.02–1.27)	782	1.41 (1.15–1.74)	0.02	4563	1.45 (1.37–1.53)	803	1.60 (1.43–1.78)	0.16
Trichloroethylene	4164	1.17 (1.04–1.32)	831	1.47 (1.20–1.81)	0.01	4359	1.40 (1.32–1.48)	864	1.57 (1.41–1.75)	0.05
White spirit	4453	1.17 (1.05–1.30)	1002	1.51 (1.26–1.81)	0.01	4650	1.53 (1.45–1.61)	1045	1.70 (1.55–1.87)	0.10
Cellulosic thinner	2286	1.07 (0.92–1.24)	326	1.30 (0.93–1.82)	0.16	2376	1.49 (1.38–1.60)	342	1.71 (1.45–2.01)	0.17
Formaldehyde	1023	1.21 (0.92–1.48)	1513	1.31 (1.12–1.54)	0.50	1095	1.44 (1.29–1.61)	1580	1.23 (1.13–1.35)	0.04
Other solvents	5614	1.17 (1.07–1.29)	4033	1.17 (1.06–1.30)	0.98	5926	1.33 (1.27–1.40)	4219	1.28 (1.21–1.35)	0.25
Paints/Varnishes	4631	1.23 (1.11–1.36)	1422	1.49 (1.28–1.73)	0.02	4856	1.48 (1.40–1.56)	1486	1.63 (1.50–1.76)	0.11
Inks/Dyes	1236	1.22 (1.01–1.46)	2334	1.34 (1.10–1.63)	0.34	1336	1.58 (1.43–1.74)	960	1.51 (1.37–1.68)	0.49

CI—Confidence Intervals; *p* inter—*p* interaction between gender and exposure. Logistic regressions or binomial negative regressions adjusted for age, BMI and smoking habits. Missing self-reported occupational exposure for current asthma: 3982 men and 5701 women. Missing self-reported occupational exposure for asthma symptom score: 4151 men and 5917 women. * Exposure to at least one solvent: gasoline, trichloroethylene, white spirit, cellulosic thinner, formaldehyde, other solvents, paints/varnishes or inks/dyes. Using the JEM, we observed no association between lifetime exposure to at least one solvent and current asthma in men (OR 0.98, 95% CI 0.91–1.05, Figure 1a), whereas the association between ever exposures to solvents and current asthma was significant in women (OR 1.12, 95% CI 1.05–1.20, Figure 1b). Using the JEM in 4 categories, no trend with a higher probability of exposure was observed (Figure 1a,b). Examining specific solvents, we observed no significant association in men, except a negative association for gasoline (Table 3), whereas lifetime exposures to gasoline, cellulosic thinner, paints and inks were positively associated with current asthma in women. Odds ratios were similar regardless of the number of solvents considered with no trend observed in men, whereas a small but significant trend was observed in women (*p* for trend: 0.001).

**Table 3:** Association between lifetime occupational exposures to solvents assessed by the JEM and current asthma or asthma symptom score.

	Current Asthma					Asthma Symptom Score				
	Men (*n* = 46,579)		Women (*n* = 45,574)			Men (*n* = 48,925)		Women (*n* = 47,450)		
	*n*	Odds Ratio (IC 95%)	*n*	Odds Ratio (IC 95%)	*p* Inter	*n*	Mean Score Ratio (IC 95%)	*n*	Mean Score Ratio (IC 95%)	*p* Inter
Non-exposed (ref.)	15,545	1	32,089	1		16,423	1	33,405	1	
At least one solvent ⁑	31,034	0.98 (0.91–1.05)	13,485	1.12 (1.05–1.20)	<0.001	32,502	1.14 (1.09–1.18)	14,045	1.10 (1.07–1.15)	0.25
1 solvent	9072	1.01 (0.92–1.10)	8065	1.12 (1.03–1.21)	0.04	9543	1.09 (1.04–1.15)	8431	1.08 (1.04–1.12)	0.77
2 solvents	3488	1.07 (0.94–1.21)	2249	1.17 (1.02–1.34)		3679	1.14 (1.07–1.23)	2337	1.18 (1.10–1.27)	
3 or more solvents	18,474	0.94 (0.87–1.02)	3171	1.08 (0.96–1.22)		19,280	1.16 (1.11–1.20)	3277	1.12 (1.06–1.20)	
*p-trend*		0.14		0.001			<0.0001		<0.0001	
Gasoline	21,564	0.92 (0.86–0.99)	3479	1.18 (1.05–1.32)	<0.001	22,606	1.14 (1.10–1.19)	3595	1.22 (1.15–1.29)	0.07
Trichloroethylene	11,184	0.93 (0.85–1.02)	1640	1.03 (0.88–1.22)	0.12	11,634	1.16 (1.11–1.22)	1694	1.10 (1.01–1.19)	0.26
White spirit	19,856	0.96 (0.89–1.04)	4250	1.10 (0.99–1.22)	0.01	20,731	1.16 (1.11–1.21)	4396	1.16 (1.10–1.22)	0.88
Cellulosic thinner	12,677	0.94 (0.87–1.03)	1125	1.29 (1.07–1.55)	<0.001	13,204	1.18 (1.13–1.24)	1167	1.24 (1.13–1.37)	0.47
Formaldehyde	4872	1.02 (0.91–1.14)	4814	1.02 (0.92–1.13)	0.96	5151	1.10 (1.04–1.17)	5022	0.94 (0.89–0.99)	<0.001
Paints/Varnishes	24,620	0.98 (0.91–1.05)	5111	1.15 (1.05–1.27)	0.001	25,723	1.16 (1.11–1.20)	5313	1.18 (1.13–1.24)	0.71
Inks/Dyes	5909	0.99 (0.89–1.10)	4609	1.18 (1.06–1.30)	0.004	6180	1.26 (1.19–1.33)	4804	1.15 (1.10–1.22)	0.01

CI—Confidence Intervals; *p* inter—*p* interaction between gender and exposure. Logistic regressions or binomial negative regressions adjusted for age, BMI and smoking habits. Missing exposure data in the JEM for current asthma: 9 men and 29 women. Missing exposure data in the JEM for asthma symptom score: 14 men and 31 women. ⁑ Exposure to at least one solvent: gasoline, trichloroethylene, white spirit, cellulosic thinner, formaldehyde, paints/varnishes or inks/dyes.

**Table 4:** Lifetime exposures to solvents and asthma symptom score stratified by ever asthmatic status.

		Asthma Symptom Score				
	Men			Women		
	*n*	Mean Score Ratio (IC 95%)	*p* Inter	*n*	Mean Score Ratio (IC 95%)	*p* Inter
Self-report *	49,206		<0.001	56,483		<0.001
Participants with ever asthma	6714	1.16 (1.09–1.25)		7623	1.18 (1.11–1.26)	
Participants without asthma	42,492	1.41 (1.36–1.47)		48,860	1.39 (1.32–1.45)	
JEM ⁑	48,925		0.03	47,450		0.59
Participants with asthma	6585	1.12 (1.05–1.20)		6334	1.08 (1.02–1.15)	
Participants without asthma	42,340	1.16 (1.12–1.21)		41,116	1.09 (1.05–1.13)	

* Exposure to at least one solvent: gasoline, trichloroethylene, white spirit, cellulosic thinner, formaldehyde, other solvents, paints/varnishes or inks/dyes. ⁑ Exposure to at least one solvent: gasoline, trichloroethylene, white spirit, cellulosic thinner, formaldehyde, paints/varnishes or inks/dyes. CI—Confidence intervals; *p* inter—*p* interaction between asthmatic status and exposure to solvent. Binomial negative regressions adjusted for age, smoking habits, and BMI.

## Data Availability

Data were obtained from the CONSTANCES group. It can be provided upon reasonable request after approval by the CONSTANCES scientific committee.

## Supplementary Materials

The following are available online at https://www.mdpi.com/article/10.3390/ijerph18179258/s1, Figure S1: Flowchart illustrating population selection, Table S1: Description of included and excluded participants, Table S2: Association between self-reported lifetime occupational exposures to solvents and current asthma or asthma symptom score in participants without exposures to welding fumes and metals dusts, Table S3: Current exposures to solvents and current asthma and asthma symptom score, Table S4: Lifetime exposure to solvents and current asthma according to age of asthma onset.
