# Supplementary material for: Occupational Exposures to Organic Solvents and Asthma Symptoms in the CONSTANCES Cohort
Source: Int J Environ Res Public Health. 2021 Sep 2;18(17):9258. doi: 10.3390/ijerph18179258 (PMC8431091; doi:10.3390/ijerph18179258)
Supplement: Supplementary file 1 [file ijerph-18-09258-s001.zip › ijerph-1312507-SI.pdf]

International Journal of
Environmental Research and Public Health

MDPI

Supplementary material

Occupational Exposures to Organic Solvents and Asthma Symptoms in the CONSTANCES Cohort

Guillaume Sit[1], Noémie Letellier[2], Yuriko Iwatsubo[3], Marcel Goldberg[4,5], Bénédicte Leynaert[1], Rachel Nadif[1], Céline Ribet[4], Nicolas Roche[1,6], Yves Roquelaure[7], Raphaëlle Varraso[1], Marie Zins[4,5], Alexis Descatha[1,7], Nicole Le Moual[1], Orianne Dumas[1]

1. Université Paris-Saclay, UVSQ, Univ. Paris-Sud, Inserm, Équipe d'Épidémiologie respiratoire intégrative, CESP, 94807 Villejuif, France ; guillaume.sit@inserm.fr (G.S), benedicte.leynaert@inserm.fr (B.L), rachel.nadif@inserm.fr (R.N), nicolas.roche@aphp.fr (N.R), raphaelle.varraso@inserm.fr (R.V), orianne.dumas@inserm.fr (O.D)
2. Herbert Wertheim School of Public Health and Human Longevity Science & Scripps Institution of Oceanography, UC San Diego, La Jolla, CA, 92093 USA; noemie.letellier@inserm.fr (N.L)
3. Santé publique France Direction Santé Environnement Travail, 94415 Saint-Maurice, France ; yuriko.IWATSUBO@santepubliquefrance.fr (Y.I)
4. Population-Based Epidemiological Cohorts Unit, INSERM UMS 11, 94807 Villejuif, France ; marcel.goldberg@inserm.fr (M.G), marie.zins@inserm.fr (M.Z), celine.ribet@inserm.fr (C.R)
5. Faculty of Medicine, University of Paris, 75006 Paris, France
6. APHP Centre – Université de Paris, Hôpital et Institut Cochin, Service de Pneumologie, Paris, 75014 France
7. UNIV Angers, CHU Angers, Univ Rennes, Inserm, EHESP, Irset (Institut de recherche en santé, environnement et travail) - UMR_S1085, F-49000 Angers, France ; alexis.descatha@inserm.fr (A.D), yves.roquelaure@univ-angers.fr (Y.R)

* Correspondence: nicole.lemoual@inserm.fr

Citation: Sit, G.; Letellier, N.; Iwatsubo, Y.; Goldberg, M.; Leynaert, B.; Nadif, R.; Ribet, C.; Roche, N.; Roquelaure, Y.; Varraso, R.; et al. Occupational Exposures to Organic Solvents and Asthma Symptoms in the CONSTANCES Cohort. *Int. J. Environ. Res. Public Health* **2021**, *18*, 9258. https://doi.org/10.3390/ijerph18179258

Academic Editor: Paul B. Tchounwou

Received: 8 July 2021
Accepted: 27 August 2021
Published: 2 Septermber 2021

Publisher's Note: MDPI stays neutral with regard to jurisdictional claims in published maps and institutional affiliations.

Copyright: © 2021 by the authors. Submitted for possible open access publication under the terms and conditions of the Creative Commons Attribution (CC BY) license (http://creativecommons.org/licenses/by/4.0/).

1. Methods

Population Job-Exposure Matrix (JEM) based on self-report

Early 2020, full job history data were available for 131,057 participants. Among them, 106,734 were working at the time of inclusion or in the 2 years prior. Among these current workers, we selected those who had available data on occupational exposures to solvents and 3-4 digit PCS-2003 ('Profession et Catégories Socio-professionnelles') job codes (n=89,134).

Our JEM has 2 axes: a "job axis" with all the existing job codes of the French PCS-2003 classification available in CONSTANCES and an "exposure axis" with 7 solvents. Separate JEMs were built in women and men.

Among all the current jobs of the 89,134 participants, we first selected all the job codes with 5 or more participants. Jobs codes with less than 5 participants were grouped with one or several similar 3- digit jobs codes to reach at least 5 participants. If after grouping the total number of participants for a 3- digit job code remained lesser than 5, we did not include all the 4 digit jobs codes concerned in the JEM.

Regarding exposures, we used self-reported current exposures which corresponded to an exposure in the two last years. We chose to use current exposures because we could link it to the current jobs and it was less prone to recall bias than past exposures. For each job we evaluated the percentage of participants who reported exposure to each solvent. Depending on these percentages, we classified for a given solvent each job into 4 categories:

0 (No exposure): percentage less than 5%
1 (Low probability of exposure): percentage from 5% to 9.9%
2 (Moderate probability of exposure): percentage from 10% to 19.9%
3 (High probability of exposure): percentage greater or equal to 20%

For all analyses with the JEM, participants exposed to "other solvents" were excluded, as we considered that this exposure was too broad to obtain valid estimates with the JEM. Thus, exposure to at least one solvent corresponded to participants exposed to gasoline, trichloroethylene, white spirit, cellulosic thinner, formaldehyde, paints/varnishes or inks/dyes. The reference category corresponded to participants never exposed to any solvents, including other solvents.

Figure S1. Flowchart illustrating population selection

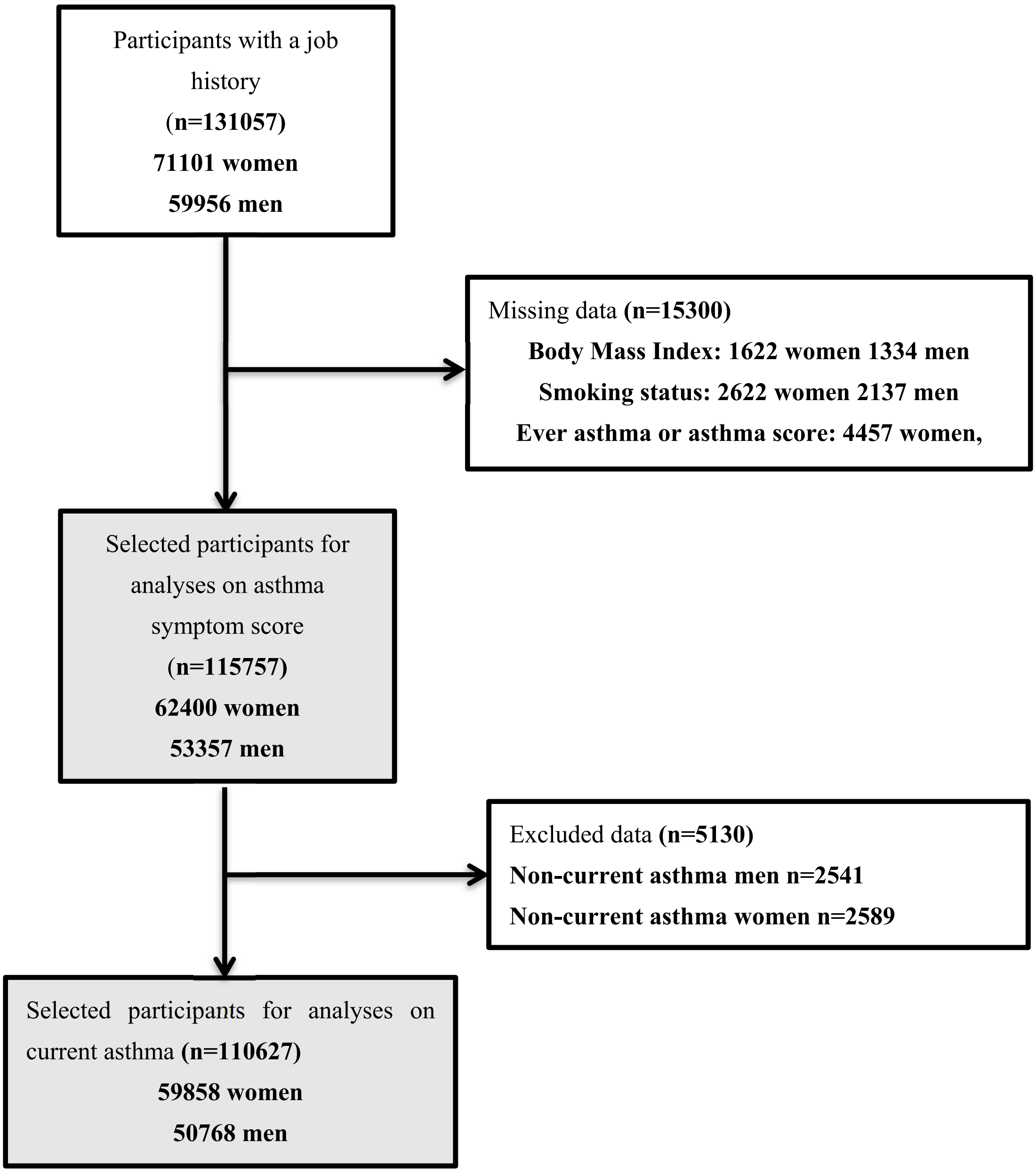

Figure S2. Questions for asthma symptoms and asthma in Constances cohort

Questions used to define the asthma symptom score in Constances:

23. Have you had wheezing or whistling in your chest at any time in the last 12 months? (Yes or No)

23.1 If yes to 23, Have you been at all breathless when wheezing noise was present? (Yes or No)

24. Have you woken up with a feeling of tightness in your chest at any time in the last 12 months? (Yes or No)

25. Have you had an attack of shortness of breath that came on during the day when you were at rest in the last 12 months? (Yes or No)

26. Have you had an attack of shortness of breath that came on following strenuous activity at any time in the last 12 months? (Yes or No)

27. Have you been woken by an attack of shortness of breath at any time in the last 12 months? (Yes or No)

Questions used to define ever and current asthma in Constances :

35. Have you ever had asthma? (Yes or No)

35.1 If yes to 35, was this confirmed by a doctor? (Yes or No)

35.4 If yes to 35, have you had an attack of asthma in the last 12 months? (Yes or No)

35.5 If yes to 35, are you currently tang any medicines including inhalers, aerosols or tablets for asthma? (Yes or No)

Table S1. Description of included and excluded participants

	Included n=115757	Missing BMI n=2956	p	Missing smoking n=4759	p	Missing asthma n=7585	p
Gender (women, %)	53.9	54.9	0.49	55.1	0.23	58.8	<0.001
Age, mean (Standard deviation)	47.2 (12.9)	46.3 (12.8)	<0.001	48.9 (12.6)	<0.001	49.7 (13.0)	<0.001
BMI, kg/m^2					0.02		<0.001
<25	57.6	-		55.4		53.9	
25 - 29.9	30.5	-		31.9		31.8	
≥30	11.9	-		12.7		14.4	
Smoking habits							
Nonsmoker	46.5	48.1	0.18	-		44.8	0.01
Current smoker	18.9	18.4		-		20.0	
Former smoker	34.7	33.4		-		35.2	
Exposures to solvents (self-report)							
At least one solvent	19.5	19.9	0.72	19.8	0.83	21.4	<0.001
Ever asthma	13.6	14.6	0.19	11.9	<0.001	-	
Current asthma	9.6	10.3	0.38	8.5	0.003	-	
Asthma symptom score							
0	68.6	68.3	0.94	67.8	0.14	-	
1	19.4	19.7		20.6		-	
≥2	12.1	12.0		11.6		-	

Results presented as percentages unless otherwise specified.
BMI – body mass index
p for Chi Square test

Table S2. Association between self-reported lifetime occupational exposures to solvents and current asthma or asthma symptom score in participants without exposures to welding fumes and metals dusts

	Current asthma					Asthma symptom score				
	Men (n=39085)		Women (n=53085)			Men (n=41095)		Women (n=55368)		
	n	Odds ratio (95% CI)	n	Odds ratio (95% CI)	p inter	n	Mean score ratio (95% CI)	n	Mean score ratio (95% CI)	p inter
Non-exposed (ref.)	28473	1	46267	1		29942	1	48251	1	
At least one solvent*	10612	1.12 (1.04-1.21)	6818	1.28 (1.18-1.38)	0.82	11153	1.33 (1.28-1.39)	7117	1.36 (1.31-1.42)	0.46
1 solvent	4627	1.04 (0.94-1.16)	4479	1.26 (1.14-1.39)		4876	1.21 (1.14-1.28)	4677	1.29 (1.23-1.36)	0.86
2 solvents	2579	1.07 (0.94-1.23)	1492	1.23 (1.05-1.44)	0.89	2712	1.33 (1.24-1.43)	1557	1.42 (1.30-1.54)	
3 or more solvents	3406	1.27 (1.13-1.43)	847	1.45 (1.18-1.77)		3565	1.51 (1.42-1.61)	883	1.61 (1.45-1.79)	
p-trend		*<0.0001*		*<0.0001*			*<0.0001*		*<0.0001*	
Gasoline	3613	1.14 (1.01-1.28)	764	1.39 (1.13-1.71)	0.24	3775	1.45 (1.36-1.54)	785	1.59 (1.43-1.78)	0.78
Trichloroethylene	3482	1.19 (1.05-1.35)	814	1.45 (1.17-1.79)	0.50	3649	1.38 (1.29-1.47)	847	1.57 (1.41-1.75)	0.49
White spirit	3711	1.19 (1.06-1.33)	978	1.49 (1.25-1.79)	0.66	3878	1.53 (1.44-1.62)	1021	1.69 (1.54-1.86)	0.33
Cellulosic thinner	1888	1.06 (0.89-1.25)	320	1.30 (0.93-1.82)	0.09	1958	1.46 (1.34-1.58)	335	1.71 (1.45-2.02)	0.73
Formaldehyde	848	1.18 (0.94-1.48)	1480	1.31 (1.11-1.54)	0.13	912	1.42 (1.26-1.60)	1547	1.24 (1.13-1.35)	0.70
Other solvents	4647	1.23 (1.11-1.36)	3945	1.15 (1.04-1.28)	0.98	4939	1.34 (1.27-1.42)	4125	1.28 (1.21-1.35)	0.72
Paints/Varnishes	3797	1.23 (1.10-1.38)	1400	1.45 (1.25-1.70)	0.70	3987	1.46 (1.38-1.55)	1462	1.62 (1.49-1.76)	0.80
Inks/Dyes	1047	1.23 (1.01-1.51)	897	1.36 (1.11-1.66)	0.97	1107	1.57 (1.41-1.74)	939	1.52 (1.37-1.69)	0.65

CI - Confidence Interval

p inter – p interaction between gender and exposure

Logistic regressions or binomial negative regressions adjusted for age, BMI and smoking habits

**Exposure to at least one solvent: gasoline, trichloroethylene, white spirit, cellulosic thinner, formaldehyde, other solvents, paints/varnishes or inks/dyes*

Table S3. Current exposures to solvents and current asthma and asthma symptom score

	Current asthma				Asthma symptom score			
	Men		Women		Men		Women	
	n	Odds ratio (IC 95%)	n	Odds ratio (IC 95%)	n	Mean score ratio (IC 95%)	n	Mean score ratio (IC 95%)
Self-report*								
Ref	34040	1	47196	1	35816	1	55683	1
Current exposure	5482	1.07 (0.98-1.18)	3523	1.15 (1.03-1.28)	5795	1.35 (1.28-1.42)	3678	1.25 (1.18-1.33)
JEM⁑								
Ref.	15545	1	22959	1	16423	1	33405	1
Current exposure	12529	0.92 (0.84-1.00)	4770	0.97 (0.87-1.08)	13187	1.07 (1.02-1.12)	4969	1.04 (0.99-1.11)

**Exposure to at least one solvent: gasoline, trichloroethylene, white spirit, cellulosic thinner, formaldehyde, other solvents, paints/varnishes or inks/dyes*

⁑ *Exposure to at least one solvent: gasoline, trichloroethylene, white spirit, cellulosic thinner, formaldehyde, paints/varnishes or inks/dyes*

CI - Confidence intervals

Binomial negative regressions adjusted for age, smoking habits, and BMI

Table S4. Lifetime exposure to solvents and current asthma according to age of asthma onset

	Men		Women	
	n	Odds ratio (IC95%)	n	Odds ratio (IC 95%)
Self-report*				
Ref	42492	1	48860	1
Childhood-onset asthma	2755	1.04 (0.96-1.14)	2636	1.17 (1.05-1.31)
Adult-onset asthma	1235	1.16 (1.03-1.31)	2145	1.48 (1.32-1.67)
JEM⁑				
Ref	42340	1	41116	1
Childhood-onset asthma	2722	0.96 (0.88-1.04)	2190	1.10 (1.00-1.20)
Adult-onset asthma	1201	0.97 (0.86-1.09)	1837	1.13 (1.02-1.25)

*Exposure to at least one solvent: gasoline, trichloroethylene, white spirit, cellulosic thinner, formaldehyde, other solvents, paints/varnishes or inks/dyes

⁑ Exposure to at least one solvent: gasoline, trichloroethylene, white spirit, cellulosic thinner, formaldehyde, paints/varnishes or inks

Childhood-onset asthma: age at asthma onset ≤16 years; adult-onset asthma: age at asthma onset > 16 years.
